# Slow-wave sleep predicts long-term social functioning in severe mental illness

**DOI:** 10.1371/journal.pone.0202198

**Published:** 2018-08-29

**Authors:** Henning Johannes Drews, Christian Dirk Wiesner, Christina Bethke-Jaenicke, Sara Lena Weinhold, Paul Christian Baier, Robert Göder

**Affiliations:** 1 Department of Psychiatry and Psychotherapy, Christian-Albrechts-University Kiel, Kiel, Schleswig-Holstein, Germany; 2 Department of Psychology, Christian-Albrechts-University Kiel, Kiel, Schleswig-Holstein, Germany; Charite Medical University Berlin, GERMANY

## Abstract

Sleep’s relevance for long-term social functioning in psychiatric disorders has been widely overlooked so far. Here, we investigate social functioning in a transdiagnostic sample of 31 patients with severe mental illness, namely schizophrenia (n = 15) or major depression (n = 16), in relation to their polysomnographic sleep characteristics 6 (± 2.4) years earlier. In addition, cognitive performance at follow-up and clinical characteristics (i.e., severity of disorder-related symptoms and number of hospitalizations between baseline and follow-up) are assessed. Multiple regression analysis results in a model with slow-wave sleep (SWS) and number of hospitalizations as significant predictors accounting for 50% (R^2^ = 0.507; p <0.001) of the variance in social functioning. SWS remains a significant predictor of long-term social functioning throughout a series of refining analyses which also identify baseline functioning as an additional significant predictor, whereas diagnosis is non-significant. Also, the effect of SWS on social functioning is not mediated by number of hospitalizations as assessed by a bootstrapped mediation analysis. We thus conclude that duration of slow-wave sleep is a powerful predictor of long-term social outcome in psychiatric disorders. Also, we discuss the relevance of verbal memory, symptom severity, and diagnostic category for social functioning. Future studies should test this finding by using a prospective design, a bigger sample, optimized predictor variables, and a more diverse set of diagnoses. Moreover, it should be explored whether or not treating sleep disturbances in psychiatric illnesses independently improves long-term social functioning.

## Introduction

Mental illnesses represent an enormous public health challenge, as they are a major contributor to the global burden of disease. In the WHO European Region, for instance, mental illnesses account for almost 40% of all years lived with disabilities and are a major reason for receiving social welfare benefits [1]. On the individual patient level, these epidemiological measures translate among others into markedly decreased social functioning. Identifying factors, which influence long-term social functioning in mental illness, is therefore of utmost importance to predict the individual course of the disease and to develop specific treatment strategies. So far, previous social functioning, cognition and premorbid education as well as clinical characteristics (i.e., number and duration of hospitalizations, diagnosis, and symptomatology) have been reported to be among the most relevant factors which influence long-term social outcome in psychiatric disorders [2–6].

As far as sleep as a predictor of social functioning is concerned, previous studies are rare. Only one polysomnographic study exists which addresses the matter by reporting a shorter REM sleep latency as a predictor of poorer one-year social outcome in schizophrenia [7]. However, sleep might be an important factor in long-term functional outcome as it is known to interact with cognition and disease severity in psychiatric illness [8]. Moreover, sleep and circadian rhythms constitute one of the five domains within the Research Domain Criteria (RDoC) initiative of the National Institute of Mental Health [9]. The RDoC concept supports sleep as a transdiagnostic factor contributing to the development and maintenance of psychiatric disorders and especially of severe mental illness [10]. For instance, sleep fragmentation and decreases in slow-wave sleep are found in many psychiatric disorders and especially in major depression and schizophrenia [11]. The transdiagnostic approach stimulated new studies with a transdiagnostic intervention for sleep dysfunction to improve functional impairment [12]. Moreover, the transdiagnostic approach to sleep and mental illness has been complemented by findings which indicate that functional outcome in mental illness depends on neuropsychological features rather than on the diagnostic category [5]. Therefore, the transdiagnostic perspective seems particularly promising when studying the relationship between sleep and social functioning in mental illness.

Here, we report on the results of a study evaluating the relevance of polysomnographic sleep parameters for social functioning in a transdiagnostic sample of severely mentally ill patients with schizophrenia or major depression approximately 6 (± 2.4) years later. Moreover, we integrate sleep, cognition, and clinical characteristics into a prediction model of social functioning and assess the mediating effects of the significant factors.

## Methods

### Sample and procedure

31 out of 84 patients suffering from either depression (n = 16) or schizophrenia (n = 15), who had participated in one of three polysomnographic sleep studies [13–15], were successfully contacted for follow-up approximately 6 years after the original sleep studies. Reasons for not being included in the follow-up study where death (n = 6), refusal to participate (n = 2), unknown current address (n = 20), and no response (n = 27). Diagnosing had taken place prior to the original studies and had been done according to ICD-10 criteria in the case of patients with schizophrenia (PwS) and according to DSM IV criteria based on a SCID 1 interview for patients with major depression (PwD). At follow-up, 8 of the 16 PwD presented with mild depressive symptoms (HAMD ≥ 7 [16]) and 4 others with a moderate symptomatology (HAMD ≥ 17 [16]). At time of the original studies (baseline), PwD were unmedicated and PwS were on a stable medication of amisulpride. Thus, pharmaceutic impact on sleep was controlled for. Data collection for the original studies took place from March 2000 to July 2006 (PwS) and from November 2005 to January 2008 (PwD). Follow-up appointments for both groups were between March 2010 and May 2012. At follow-up, the participants were interviewed with regards to their current social situation and disease severity and underwent testing for several cognitive domains. To ensure comparability with the baseline studies, cognitive tests at follow-up took place in the early evening (except for one case). Sample characteristics are displayed in Table 1. The results were then analyzed by direct comparison of diagnostic groups and by building regression models with cognition, sleep, and disease severity as independent variables and social functioning as the dependent variable. The study design was approved by the ethics committee of Kiel University and written informed consent was obtained.

**Table 1:** Sample characteristics regarding demographic and epidemiological measures, sleep parameters, disease severity, and cognition/education.

	Overall sample	Schizophrenia	Depression	Schizophrenia vs. Depression
N (male/female) =	31 (11/20)	15 (7/8)	16 (4/12)	
	**Mean ± SD**	**Mean ± SD**	**Mean ± SD**	**P value** (t-tests, two-tailed)
Age [years]	37.0 ± 6.7	37.9 ± 5.6	36.3 ± 7.6	0.505
Time to follow-up [years]	6.0 ± 2.4	7.5 ± 2.6	4.7±0.8	**<0.001**
Social functioning at follow-up [SF-Score]	6.0 ± 1.8	4.9 ± 1.9	6.9±1.2	**<0.002**
Social functioning at baseline [SF-Score]	6.3 ± 1.4	5.4 ± 1.3	7.1 ± 1.0	**<0.001**
**Sleep at baseline**	**Mean ± SD**	**Mean ± SD**	**Mean ± SD**	**P value**
Total sleep time [min]	393.7 ± 67.3	400.4 ± 72.4	387.4 ± 63.9	0.601
Sleep efficiency [%]	84.0 ± 10.5	83.2 ± 8.9	84.8 ± 12.0	0.675
N1 sleep [min]	44.8 ± 32.0	53.4 ± 40.6	36.7 ± 19.1	0.162
N2 sleep [min]	210.6 ± 43.5	214.4 ± 49.6	207.0 ± 38.3	0.647
SWS [min]	51.9 ± 32.1	40.8 ± 28.7	62.3 ± 32.4	0.059
REM sleep [min]	86.4 ± 32.8	91.8 ± 33.2	81.4 ± 32.6	0.387
REM latency [min]	86.0 ± 37.8	83.6 ± 31.4	88.4 ± 44.4	0.737
**Clinical characteristics**	**Mean ± SD**	**Mean ± SD**	**Mean ± SD**	**P value**
Relative symptom severity at baseline [normalized T-values]	67.4 ± 22.8	86.9 ± 17.6	49.1 ± 3.1	**<0.001**
Relative symptom severity at follow-up [normalized T-values]	46.5 ± 11.5	54 ± 11.4	39.4 ± 5.4	**<0.001**
No. of hospitalizations between baseline and follow-up	3.5 ± 9.0	6.5 ± 12.4	0.7 ± 1.2	0.093
**Cognition/Education**	**Mean ± SD**	**Mean ± SD**	**Mean ± SD**	**P value**
Education [years at school]	11.5 ± 1.7	11.3 ± 1.9	11.6 ± 1.5	0.638
Rey-Osterrieth Complex Figure at follow-up [no. of correct recalls]	21.8 ± 7.2	18.4 ± 6.9	25.1 ± 6.1	**0.009**
Trail Making Test B at follow-up [sec]	74.8 ± 53.6	91.5 ± 66.2	58.2 ± 31.2	0.093
Verbal memory at follow-up [no. of correctly memorized words across all runs]	52.7 ± 9.8	48.7 ± 9.8	56.6 ± 8.3	**0.025**

Social functioning score represents a sum score of employment situation (regular employment [3], supported employment [2], unemployment [1], disability pension [0]), living arrangement (independent [3], partially institutionalized [2], and fully institutionalized [1]), and partnership status (having a partner [2] or not [1]).

### Measures

#### Sleep parameters

Sleep data were derived from the sleep laboratory based polysomnographic measurements of the baseline studies. These measurements comprised electroencephalography (EEG; C3-A2 and C4-A1), electrooculography (EOG) and submental electromyography (EMG). Sleep stages were manually scored according to the Rechtschaffen and Kales criteria [17] by a blinded and experienced rater. Additionally, respiratory measures and EMG of the anterior tibial muscle were obtained during the first night. None of the patients showed signs of a sleep-related movement or breathing disorder as defined by an apnea-hypopnea index or a periodic-limb-movement-arousal index of more than 5/h. To control for the first night effect and interventions, only sleep data of non-intervention second nights were included in the present analysis. Total sleep time, sleep efficiency, REM latency as well as duration of N1 sleep, N2 sleep, slow-wave sleep (SWS), and REM sleep were used for further analysis.

#### Clinical characteristics

Disorder-specific symptoms of PwD were assessed by the 21-item version of the Hamilton Rating Scale for Depression (HAMD) [18]. For PwS the Positive and Negative Symptoms Scale (PANSS) for schizophrenia [19] was completed (for original values see S1 Table). To be able to include both disorder-specific scales into a transdiagnostic analysis, we transformed the individual scores of each patient into standardized T-values (M = 50, SD = 10) by using normative data from either Hamilton [18] or Kay et al. [19]. Since Kay et al. [19] only provide normative data for the PANSS subscores (negative symptoms, positive symptoms, general psychopathology) and not for the sum score, we transformed the general psychopathology subscore only. This procedure was pursued as a large study in PwS reports the PANSS general psychopathology subscale to be a significant predictor of occupational functioning, while neither the negative symptoms nor the positive symptoms subscale were significant [20]. Moreover, we could reproduce this finding in our own sample: when building regression models with the respective PANSS subscales as predictors, general psychopathology is the only significant predictor variable for social functioning at baseline (p<0.001) and follow-up (p = 0.001) (Table A and Table B in S2 Table).

It is of note that the standardized values do not allow for a direct comparison of individuals belonging to different diagnostic groups. They allow for an indirect comparison via the respective norm groups and serve as a uniform scale accounting for intragroup variance in disorder-specific symptom severity. We additionally use diagnosis as a variable to account for intergroup variance.

In addition to the disorder-specific scales, we also collected the number of hospitalizations to a psychiatric hospital between baseline and follow-up as an additional marker for disease severity.

#### Cognition and education

We assessed executive functioning, visuospatial memory, and verbal memory at follow-up. Tests employed were the Trail Making Test part B (TMT-B) [21], the Rey-Osterrieth Complex Figure Test (ROCF) [22], and the German version of the Rey Auditory-Verbal Learning Test (VLMT) [23]. Outcome measures were time needed for completion of the TMT-B, performance in the delayed recall (approximately 20–30 minutes after the learning session) in the ROCF, and sum of the correctly memorized words of all runs in the VLMT. Due to the heterogeneity of cognitive tests in the baseline studies, we could not include baseline cognitive parameters in the present work. In addition, we compiled the number of years completed at school.

#### Functional outcome

Social functioning is a broad concept. Most established scales for assessing social functioning already represent a trade-off by focusing on specific domains of functioning or disorders. Therefore, we decided to rely on a basic concept of social functioning which nevertheless includes the central hallmarks of social functioning and could also be easily assessed in an ordinary clinical setting. Thus, to evaluate social functioning, employment status, living arrangement and partnership status were assessed at follow-up. The employment status included regular employment (3), supported employment (i.e., in a therapeutic work project) (2), unemployment (1), or receiving disability pension (0). The living arrangement comprised either living independently (3), partially institutionalized (i.e., in a residential community with limited (i.e. once a day) access to therapeutic care staff) (2), or fully institutionalized (i.e., in a residential community with 24/7 access to therapeutic care staff) (1). The partnership status consisted of having a partner (2) or not (1).

This operationalization of social functioning was based on Hofer et al. [24]. The three variables were then merged into one single social functioning sum score.

### Statistical analysis

To compare the diagnostic groups, age, time to follow-up, social functioning as well as markers of sleep, disease severity, and current cognition/education were compared using two-tailed unpaired t-tests (schizophrenia vs. depression).

To assess the explanatory power of past sleep parameters, present cognition, and clinical characteristics with respect to social functioning we used a three-step approach. First, we developed separate exploratory linear regression models for each domain (cognition, clinical characteristics, and sleep) by using a stepwise selection of variables. The variable selection was based on the variables’ predictive ability towards social functioning.

The “cognition” regression model included the total amount of memorized words in VLMT, performance in the delayed recall condition of the ROCF approximately 20–30 minutes after the learning session, the completion time of the TMT-B, and years of education. The “clinical characteristics” regression model comprised the diagnosis, symptom severity at baseline, symptom severity at follow-up, and number of hospitalizations to a psychiatric hospital between baseline and follow-up. The sleep domain was covered by two explorative models, one of which contained duration of sleep stages (N1, N2, SWS, and REM sleep), and the other consisted of additional sleep parameters having been reported to be altered in mental illness [8,11], namely total sleep time, sleep efficiency, and REM latency.

The second step was to build an integrative linear regression model by using the significant predictors of the domain specific exploratory models. Third, we conducted a series of refining analyses to test the robustness of our results in view of additional factors such as follow-up interval or baseline functioning, to gain a better understanding of each domain’s relevance, and to test mediating effects. To this end, we ran additional linear regression models on the whole study population or on PwD and PwS separately. Also, we conducted a bootstrapped mediation analysis to assess whether the significant predictors of the final model which were derived either from the cognition domain or the disease characteristics domain would mediate the effect of the sleep variable on social functioning. The mediation hypothesis draws on findings that sleep influences the course of disease in PwD and PwS [8] as well as being important to cognitive processes [25,26]. This holds particularly true for SWS and declarative memory [27]. Moreover, course of disease and cognition are both known to impact social functioning [4–6].

All analyses were done in SPSS (Version 23) in which the algorithm for stepwise regression model building uses both, the probability to enter (*p* ≤ 0.05) and probability to remove (*p* ≥ 0.1) a predictor. First, the variable with the smallest probability of the F-value is entered as a predictor into the equation. Next, the variable with the smallest probability of the F-value of the remaining variables is entered. A predictor already entered is removed if inclusion of another predictor results in a p-value larger than the threshold. The algorithm is repeated until no more variables are eligible for removal or inclusion. For the bootstrapped mediation analysis the SPSS-plugin Process by Hayes was used [28].

## Results

Descriptive statistics and comparisons between diagnostic groups are given in Table 1. PwD differ significantly from PwS by having a shorter time to follow-up, lower symptom severity at baseline and follow-up, better performance in ROCF and VLMT, as well as a higher social functioning at baseline and follow-up. Age, sleep parameters, number of hospitalizations between baseline and follow-up, years of scholarly education, and performance in the TMT-B did not differ significantly between diagnostic groups. Also, both diagnostic groups showed highly significant decreases of symptom load across all scales from baseline to follow-up (all ps < 0.002). The original HAMD and PANSS values including PANSS subscales are given in S1 Table.

### Exploratory regression models

**Fig 1:**
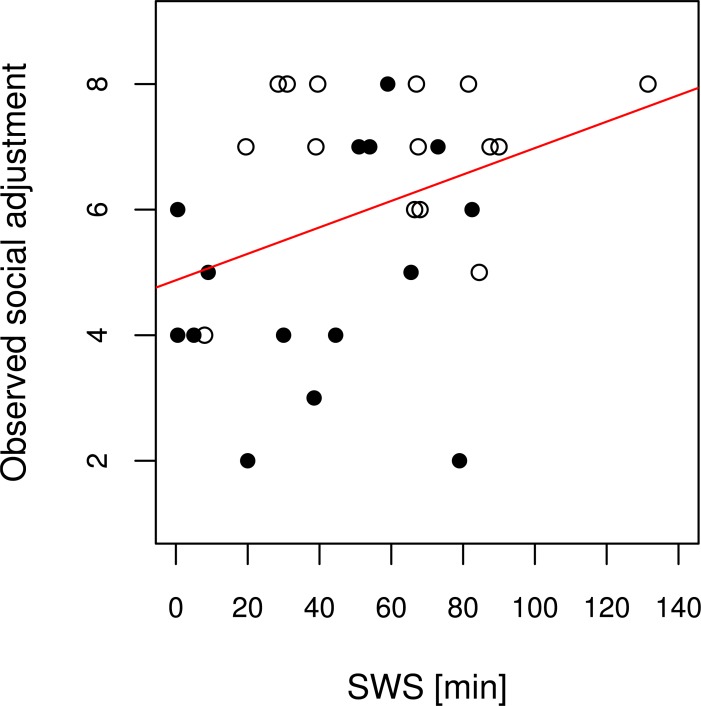
Regression plot of the exploratory sleep regression model. Stepwise variable selection of sleep stages (N1 sleep, N2 sleep, SWS, REM sleep) results in a significant regression model (p = 0.042; adjusted R^2^ = 0.106) with SWS as the only significant predictor. The given scatterplot represents this positive linear relationship (β = 0.368) between SWS and social functioning. Black dots represent PwS, black-edged white dots represent PwD. The red line represents the regression line of the respective model.

The detailed results of the exploratory regression models with significant results are given in Table 2. In the sleep models, the only significant predictor of social functioning was the amount of SWS (Fig 1). N1 sleep, N2 sleep, and REM sleep remained non-significant (all ps > 0.360). This was also true for the second sleep regression model including total sleep time, sleep efficiency, and REM latency (adjusted R^2^≈0; p(model) = 0.781; all ps(predictors) > 0.454).

**Table 2:** Explorative multiple regression analyses.

Model	Variables	Standardized beta coefficient	*p*-value
1. Sleep parameters	SWS	0.368	**0.042**
(R^2^ = 0.106; p = 0.042)	N1 sleep	-0.103	0.613
	N2 sleep	0.001	0.993
	REM sleep	-0.195	0.360
2. Disease severity	Hospitalizations	-0.356	**0.021**
(R^2^ = 0.423; p<0.001)	Symptom severity (baseline)	-0.477	**0.003**
	Symptom severity (follow-up)	-0.257	0.231
	Diagnosis	0.128	0.633
3. Cognition/Education	VLMT	0.394	**0.031**
(R^2^ = 0.125; p = 0.031)	TMT-B	-0.059	0.749
	ROCF	-0.071	0.749
	Education	0.021	0.911

Linear regression models using social functioning as the dependent variable. Variable selection: stepwise. SWS = Slow-wave sleep; VLMT = Rey Auditory-Verbal Learning Test (German version); TMT-B = Trail Making Test (Part B); ROCF = Rey-Osterrieth Complex Figure. Not displayed is the second sleep model with REM latency, total sleep time, and sleep efficiency as no significant predictor emerged from this model. Reported are adjusted R^2^.

The regression model of cognition showed the performance in verbal memory at follow-up as a significant predictor of social functioning, whereas TMT, ROCF and years at school (all ps > 0.749) were not significant predictors. Finally, the number of hospitalizations and symptom severity at baseline were significantly associated with social functioning in the disease severity model. Diagnosis and symptom severity at follow-up, were not significant predictors of social functioning at follow-up (all ps > 0.231).

### The integrative regression model

The significant parameters of the exploratory regression models were introduced into a final multiple linear regression model (Table 3). In total, the predictors SWS at baseline and number of hospitalizations between baseline and follow-up remained significant predictors. Higher amounts of SWS and less hospitalizations predicted social functioning at follow-up. Verbal memory at follow-up and baseline symptom severity were no longer significant predictors of social functioning. Altogether, the final regression model accounted for approximately half of the variance in social functioning as reflected by employment status, living arrangement, and relationship status (adjusted R^2^ = 0.507).

**Table 3:** Final multiple regression model using follow-up social functioning as the dependent variable.

Model	Variables	Standardized beta coefficient	*P*-value
Final	SWS	0.317	0.035
(R^2^ = 0.507; p < 0.001)	VLMT	0.224	0.160
	Hospitalizations	-0.482	0.003
	Symptom severity (baseline)	-0.209	0.229

SWS = Slow-wave sleep; VLMT = Rey Auditory-Verbal Learning Test (German version). Reported is the adjusted R^2^.

**Fig 2:**
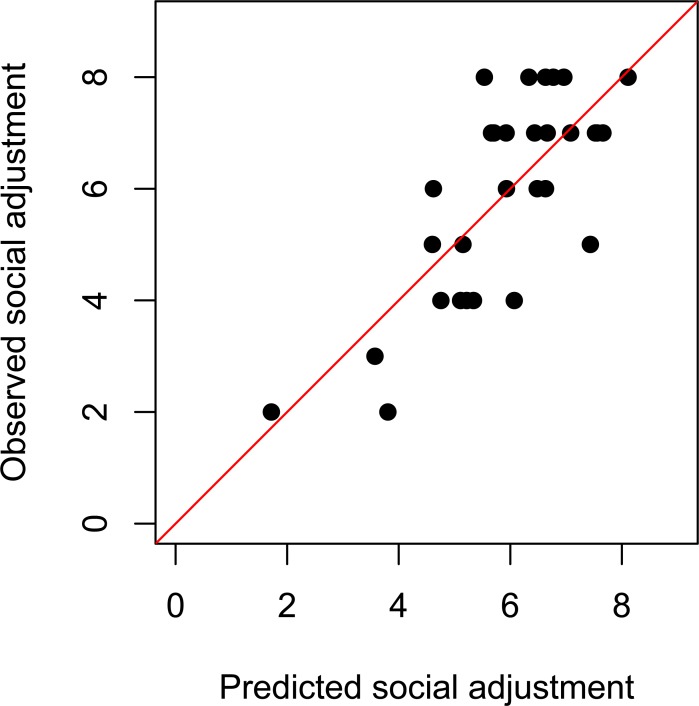
Regression plot of the final regression model. Regression plot of the final regression model (adjusted R^2^ = 0.507), including SWS (β = 0.317; p = 0.035) and hospitalizations (β = -0.482; p = 0.003) as significant predictors. Verbal memory (VLMT; β = 0.224; p = 0.160) and symptom severity are non-significant predictors of the model (β = -0.209; p = 0.229).

Fig 2 shows the regression graph of observed over predicted social adjustment.

### Refining analyses

The objective of the following analyses was to evaluate how robust the integrative model is. We were especially interested whether SWS remains a significant predictor in the model if we add other potentially confounding variables as predictors.

#### Adjusting for follow-up interval, baseline functioning, and outlier in no. of hospitalizations

One subject was hospitalized 50 times compared to 8 times or less in the remainder of the sample (see S1 Dataset). Also, there was a wide variation in time to follow-up with significant differences between diagnostic groups (Table 1) and baseline functioning has been reported to be a predictor of long-term functioning [6]. Therefore, we computed three additional regression models representing modifications of the final regression model in which either the outlier was excluded or time to follow-up or baseline functioning were included as predictor variables. All resulting models were highly significant (all ps <0.001). Baseline functioning was an additional significant predictor of follow-up social functioning (p = 0.033, β = 0.378) and the respective model showed an increased adjusted R^2^ of 0.577 as compared to the integrative model (R^2^ = 0.507). With a p-value of 0.679, a β of -0.029, and R^2^ of 0.486 the model including time to follow-up did not provide additional explanatory value. The same holds true for the model in which the outlier was removed from the sample (R^2^ = 0.427). In this model, however, significance of hospitalizations was reduced to p = 0.086 (β = -0.278). SWS remained a significant predictor of social functioning throughout all models (all βs > 0.280; all ps<0.046). The models are given in S3A–S3C Table.

#### Relevance of diagnostic categories

To test the relevance of diagnosis and the transdiagnostic approach on the level of the integrative, final regression model we entered the diagnosis variable into the final model. While the resulting model was significant (p = 0.001; adjusted R^2^ = 0.487) and SWS and hospitalizations remained significant predictors of social functioning (all ps < 0.044; β(hosp.) = -0.481; β(SWS) = 0.315), diagnosis was not significant (p = 0.961; β = 0.012). A similar result of a non-significant diagnosis variable (p = 0.743; β = -0.078) and preserved significance of the formerly significant predictors, including SWS, (all ps < 0.048; β(SWS) = 0.288; β(hosp) = -0.431; β(basel. funct.) = 0.387) is found when inserting diagnosis into the refined model including baseline social functioning (p(model) < 0.001; adjusted R^2^ = 0.561).

Application of the integrative regression model to the schizophrenia subgroup results in an overall significant model (p = 0.021) with an adjusted R^2^ of 0.514. SWS at baseline (β = 0.460; p = 0.048), and hospitalizations between baseline and follow-up (β = -0.790; p = 0.004) are significant predictors of social functioning. Verbal memory at follow-up (β = 0.482; p = 0.057) is by trend significant. The model for PwD, however, is non-significant (p = 0.762) with no significant predictor (all ps > 0.253). The detailed models are given in S3D–S3G Table.

#### Mediation analysis

A priori, we hypothesized that clinical characteristics and cognitive markers could mediate an effect of SWS on long-term social functioning. The only significant predictor from the cognitive or the clinical characteristics domain was number of hospitalizations. Therefore, we computed a bootstrapped mediation analysis entering SWS as the predictor, social functioning as the criterion and hospitalizations as potential mediator. The analysis showed a significant positive direct effect of SWS on social functioning (effect = 0.025; 95%-CI: 0.008–0.041). The indirect effect of SWS via hospitalizations on social functioning did not reach significance (effect = -0.004; CI: -0.016–0.010). Thus, hospitalizations did not mediate an indirect effect.

**Fig 3:**
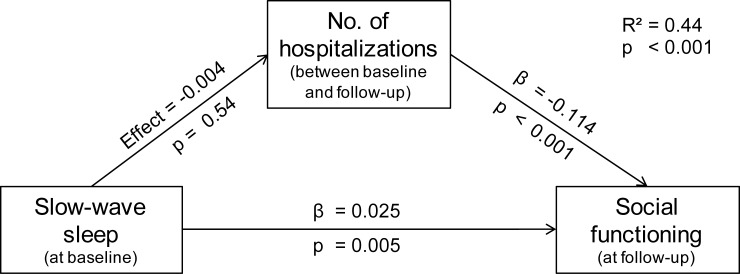
Bootstrapped mediation analysis. No significant mediating effect of number of hospitalizations on social functioning was found. Reported is the adjusted R^2^.

In summary, the mediation analysis confirmed that slow-wave sleep itself predicts functional outcome independently of number of hospitalizations. Note, that this statistical association is only a prerequisite for a causal relationship but does not constitute sufficient proof for causation. Further statistics are reported in Fig 3.

## Discussion

The present study integrates sleep into a prediction model of social outcome in psychiatric disorders. Baseline SWS and number of hospitalizations are significant predictors of social functioning accounting for approximately half of the variance. Follow-up verbal memory and baseline symptom severity are non-significant parts of the integrative model. The final model can be further enhanced by using baseline social functioning as an additional predictor variable. Diagnostic category is of secondary importance.

### Sleep and social outcome in mental illness

Long-term social and vocational outcome in psychiatric disorders has mostly been investigated without considering sleep. The present study reports two relevant findings regarding sleep and social functioning. First, SWS is the only significant sleep parameter to predict long-term social functioning. Sleep duration, sleep efficiency, other sleep stages, and REM latency do not have a significant predictive capacity. The lacking effect of REM latency on social functioning contradicts findings of the only other previous work to include polysomnographic sleep data in the study of social outcome in mental illness [7]. This study reports increased REM onset latency to be associated with improved social functioning, with no relationship found between SWS and social functioning. It is, however, noteworthy that the effect of REM latency was weak and only by trend significant for the whole sample. The different findings of the two studies assessing sleep and social functioning might be explained by differences between the studies regarding follow-up period, which was markedly shorter in the study by Goldman et al. [7]. Also, a different measure of social functioning (the Strauss-Carpenter scale [29]) was employed. The Strauss-Carpenter scale includes hospitalizations and symptomatology as subscales of social functioning [29]. In the present study we subsume these items under the domain “clinical characteristics” rather than “social functioning”. Therefore, the two measures of social functioning and the resulting studies seem hardly comparable.

Regarding sleep parameters beyond REM latency and SWS, a lacking body of literature on direct effects of other sleep measures on social functioning precludes a comparison of our findings to previous works. Nevertheless, since the special role of SWS in hippocampal, declarative memory consolidation is well established (for a recent review on SWS see [27]) as is the importance of declarative verbal memory for social functioning [4], it is very plausible that patients with comparably intact slow wave activity have a higher level of functioning.

The second important finding regarding sleep and social functioning is that SWS bears a predictive capacity of follow-up social functioning which goes beyond the predictions based on baseline functioning (and number of hospitalizations). While the importance of previous functioning–either premorbid or during an earlier clinical stage–is established [6,30], our finding of an additional predictive capacity of SWS might indicate an independent long-term effect of SWS on social functioning. The mechanisms underlying the additive effect of SWS remain to be elucidated. Nevertheless, two aspects are conceivable. The first is the relevance of SWS for cognition and memory formation [27]. This mirrors an underlying sleep-related cerebral plasticity to which SWS is central [31,32]. On a social functioning level, these SWS-dependent capacities might translate into the ability to maintain social structures despite of a mental condition and to reestablish them after they have been disrupted in an acute illness episode. Likewise, the influence of SWS on clinical characteristics might be an important mechanism. In this context, sleep has been reported to positively impact symptom load, relapses, and treatment response in different mental illnesses including major depression [8]. In the following, cognition and clinical characteristics are discussed regarding their relation to social functioning.

### Cognition and social functioning

A variety of previous studies has found cognition to be related to social functioning across a number of psychiatric disorders including major depression [5,33], bipolar disorder [2,5], and schizophrenia [3–5]. However, the relevant cognitive domains and the prediction periods vary across studies. As far as cognitive performance and social functioning are assessed at the same point in time, Simonsen et al. [34] report a significant association of work performance with working memory and verbal memory among others. Moreover, a particular relevance of verbal memory for maintaining employment in schizophrenia has been reported [35]. In accordance with these previous findings, the present study shows verbal memory to be a significant predictor of social functioning in the regression model of cognitive markers. This is in line with a review by Green et al. which found verbal memory to be the best neurocognitive predictor for social functioning [4]. On the level of the final integrative regression model however, follow-up verbal memory is a non-significant predictor for follow-up social functioning. This lacking significance might be due to our rather small sample size. Also, SWS at baseline and number of hospitalizations might be sufficient to explain the variance in social functioning. Thus, verbal memory would not add additional explanatory value. More importantly, however, there is one major difference between the present work and previous works studying the interrelationship of SWS and cognition. The present study compares SWS at baseline with verbal memory at follow-up about six years later, whereas previous research usually looked at overnight effects connecting the amount of SWS to memory consolidation during the same night (i.e. [13–15], for a review on sleep and memory formation see [26]). In short, manifold relations between cognition and social functioning have been reported with verbal memory being of particular importance. While this could be (partly) reproduced in the present study, a more elaborate future study should be conducted to profoundly address the interplay of SWS, cognition, and social functioning.

### Clinical characteristics and social outcome–Does diagnosis matter?

In the current work, number of hospitalizations, symptom severity, and diagnostic category are assessed regarding their relevance for long-term social functioning. Each of these clinical characteristics has been reported to contribute to social functioning in psychiatric disorders [2,3,6,36]. In the present study, only the general psychopathology subscale of the PANSS predicts social functioning, whereas positive or negative symptoms do not. This is in line with a previous study by Giugiario et al. which moreover overlaps with ours by reporting a high relevance of verbal memory for vocational outcome [20]. Regarding other markers of disease severity, we here report, that the number of hospitalizations between baseline and follow-up predicts social functioning significantly in both the explorative “clinical characteristics” model and the final integrative model. This reproduces previous reports [6]. However, when adjusting for an outlier with an extraordinary high number of hospitalizations, the effect is markedly reduced, but remains by trend significant.

Regarding the relevance of diagnostic category for social functioning, we found significant differences between PwS and PwD when comparing the diagnostic groups directly by using t-tests. Also, applying the final integrative regression model to the PwD subgroup nullifies the effect, which might indicate that the observed effect in the whole sample is driven by the schizophrenia group. However, when the diagnostic category is used as a predictor variable in the regression models it does not reach significance, neither at level the of the explanatory models nor when inserted into the final model. Therefore, it seems as if diagnosis might work as a proxy for social functioning but in fact does not stand up to a more detailed analysis. This finding supports the notion of Lee et al. [5] who reported an insufficiency of traditional diagnostic categories to predict long-term social outcome. Moreover, this ambiguous relevance of the particular diagnoses for social functioning as reported here seems to mirror a similar phenomenon regarding sleep disturbances in mental illnesses. On the one hand, sleep disturbances in mental illnesses have traditionally been conceptualized as disorder-specific symptoms and as being associated with a worse course of the underlying psychiatric disorders [8]. On the other hand, sleep disturbances in mental illness have been argued to be independent of the underlying diagnosis and be either transdiagnostic [10] or partly transdiagnostic as well as rather influenced by co-morbidities. The latter has been argued to be particularly true for changes in SWS [11].

### Mediating effects of clinical characteristics

Building on our hypothesis that SWS could impact cognition and clinical characteristics which in turn would define social outcome, we tested whether the number of hospitalizations would serve as a marker for a more complex clinical course and mediate the effect of SWS on social functioning. Since previous works have found both, an impact of sleep on course of the disease [8] and an effect of hospitalizations on social functioning [6] a mediation of an indirect effect by number of hospitalizations seemed likely.

However, mediation analysis did not support our mediation hypothesis. This might be explained by a potential systematic error of using number of hospitalizations as a proxy for disease severity. The direction of the relationship between social functioning and hospitalizations is unclear. Possibly, lower social functioning result in lower social support leading to earlier and more frequent hospitalizations.

We did not test any other possible mediator due to the lack of other significant predictors matching our initial mediation hypothesis and due to the small sample size, which precluded more complex analyses.

It seems however likely that for instance verbal memory could mediate such an effect. SWS has been reported to be especially important for memory consolidation in healthy individuals as well as in psychiatric patients [13–15,25,26]. In addition, the relation between verbal memory and social functioning is well established [4,20]. Thus, the relationship between SWS, cognition, and social functioning should be addressed in the future.

## Conclusion, limitations, and outlook

The present study shows the importance of SWS in predicting long-term social outcome in a transdiagnostic sample of patients with major depression and schizophrenia. This predictive capacity is independent of baseline functioning and number of hospitalizations which are additional significant predictors in a multiple regression model, whereas diagnosis is not a significant predictor. Besides these novel and important findings, the study is limited in some respects. The statistical approach (multiple linear regression analysis) includes a rather low number of cases per predictor variable and a comparably high number of analyses. Regarding the former, there is recent evidence that a low number of cases per variable does not compromise model validity per se [37]. However, it results in a decreased statistical power representing an increased risk of type two errors. Therefore, our study underlines the importance of SWS for long-term social functioning but might report falsely non-significant predictors. This is particularly true for verbal memory and symptom severity which are significant predictors of social functioning in the exploratory models but lose their significance in the final model. Thus, we suggest interpreting our results not as devaluating cognition or symptom severity as predictors of social functioning, but as making the case for SWS in that respect. The other statistical limitation, namely the multiple testing, increases the risk of type one errors. The fact that our results are in line with existing previous works (i.e., verbal memory [4], baseline functioning [6], hospitalizations [6], symptom severity [20]) or are very robust in a variety of different models (as is the case for SWS) increases plausibility of the presented results. Nevertheless, we cannot exclude that some of the significant effects are due to type one errors. Moreover, the assessment of cognition is limited in two ways. First, the heterogeneity of tests employed in the baseline studies precludes inclusion of baseline cognitive measures. Second, we only assessed a limited (albeit well-considered) set of cognitive domains and therefore potentially miss out effects of potentially relevant domains such as attention [5], working memory [5,34], or social cognition [38]. This limits the in-depth elucidation of the interaction of sleep, cognition, and disease severity over time.

Likewise, it is important to note that social functioning is a complex construct and its assessment often comes along with emphasizing aspects of it. The present study uses a basic concept of social functioning consisting of three fields: employment status, living arrangement, and partnership status. However, the use of another operationalization of social functioning might lead to other results. Another limitation is, despite not being a significant predictor for social functioning, the heterogeneity in follow-up intervals. And finally, the low response-rate might introduce a survivorship bias to the present study.

Despite these limitations, the present work provides important new results as it highlights the role of SWS in the long-term prediction of social outcome in mental illness. It further supports the notion of a very close relationship between sleep and the course of psychiatric disorders.

Future studies dealing with social outcome in psychiatric disorders should assess the interplay of sleep, cognition, clinical characteristics and functioning over time by using a more suitable marker for disease severity (i.e. a clinical global impression score) and a bigger sample. A prospective study design including equal follow-up intervals, identical tests at all time points, and a healthy control group should be used. Moreover, it should be tested whether or not treating sleep disturbances in psychiatric disorders independently of the mere disorder-specific symptomatology yields additional benefits for the long-term social outcome.

## Supporting information

S1 Dataset Original data. (XLSX)

S1 Table Disorder specific symptom severity at baseline and follow-up. Diagnostic groups show a marked decrease in symptom severity from baseline to follow up.
HAMD = Hamilton Rating Scale for Depression; PANSSpos = positive symptom subscale of the Positive and Negative Symptoms Scale for Schizophrenia; PANSSneg = negative symptom subscale of the PANSS; PANSSgen = general psychopathology subscale of the PANSS.
(DOCX)

S2 Table Regression models of PANSS-subscores with social functioning as dependent variable. **A. At baseline**.
**B. At follow-up**.
Generals psychopathology but neither negative symptoms nor positive symptoms predict social functioning at baseline (A) or follow-up (B). PANSSneg = negative symptom subscale of the PANSS; PANSSpos = positive symptom subscale of the Positive and Negative Symptoms Scale for Schizophrenia; PANSSgen = general psychopathology subscale of the PANSS. Reported are adjusted R^2^.
(DOCX)

S3 Table Refining regression models. **A. Integrative regression model under exclusion of an outlier in numbers of hospitalizations**.
Exclusion of the outlier reduces the predictive value of the hospitalizations variable. Slow-wave sleep (SWS) remains a significant predictor. The whole model has a lower R^2^ than the original integrative regression model (R^2^ = 0.427 vs. R^2^ = 0.507). SWS = Slow-wave sleep; VLMT = Rey Auditory-Verbal Learning Test (German version). Reported is the adjusted R^2^.
**B. Time to follow-up is added to the integrative regression model as predictor variable**.
Time to follow-up is not a significant predictor of social functioning. Slow-wave sleep (SWS) remains a significant predictor. The whole model has a lower R^2^ than the original integrative regression model (R^2^ = 0.486 vs. R^2^ = 0.507). SWS = Slow-wave sleep; VLMT = Rey Auditory-Verbal Learning Test (German version). Reported is the adjusted R^2^.
**C. Baseline social functioning is added to the integrative regression model as predictor variable**.
Baseline social functioning is a significant predictor of social functioning. Slow-wave sleep (SWS) remains a significant predictor. The whole model has a higher R^2^ than the original integrative regression model (R^2^ = 0.577 vs. R^2^ = 0.507). SWS = Slow-wave sleep; VLMT = Rey Auditory-Verbal Learning Test (German version). Reported is the adjusted R^2^.
**D. Diagnosis is added to the integrative regression model as predictor variable**.
As in the explorative clincal characteristics model, diagnosis is not a significant predictor of social functioning when added to the integrative regression model. Slow-wave sleep (SWS) remains a significant predictor. The whole model has a lower R^2^ than the original integrative regression model (R^2^ = 0.487 vs. R^2^ = 0.507). SWS = Slow-wave sleep; VLMT = Rey Auditory-Verbal Learning Test (German version). Reported is the adjusted R^2^.
**E. Diagnosis is added to the refined regression model including baseline functioning as predictor variable**.
As in the explorative disease characteristics model, diagnosis is not a significant predictor of social functioning when added to the refined integrative regression model also including baseline social functioning. Slow-wave sleep (SWS) remains a significant predictor. The whole model has a lower R^2^ than the refined integrative regression model (R^2^ = 0.561 vs. R^2^ = 0.577). SWS = Slow-wave sleep; VLMT = Rey Auditory-Verbal Learning Test (German version). Reported is the adjusted R^2^.
**F. Application of the integrative regression model to the depression group only**.
The integrative regression model is no longer significant, if applied to the depression subgroup alone. SWS = Slow-wave sleep; VLMT = Rey Auditory-Verbal Learning Test (German version). Reported is the adjusted R^2^.
**G. Application of the integrative regression model to the schizophrenia group only**.
Application of the integrative regression model to the schizophrenia subgroup results in a significant model with SWS and no. of hospitalizations as significant predictors. Verbal memory tends towards significance. SWS = Slow-wave sleep; VLMT = Rey Auditory-Verbal Learning Test (German version). Reported is the adjusted R^2^.
(DOCX)
