# Supplementary material for: Slow-wave sleep predicts long-term social functioning in severe mental illness
Source: PLoS One. 2018 Aug 29;13(8):e0202198. doi: 10.1371/journal.pone.0202198 (PMC6114721; doi:10.1371/journal.pone.0202198)
Supplement: S1 Table — Diagnostic groups show a marked decrease in symptom severity from baseline to follow up. HAMD = Hamilton Rating Scale for Depression; PANSSpos = positive symptom subscale of the Positive and Negative Symptoms Scale for Schizophrenia; PANSSneg = negative symptom subscale of the PANSS; PANSSgen = general psychopathology subscale of the PANSS. (DOCX) [file pone.0202198.s002.docx]

**S1 Table. Disease specific symptom severity.**

| **Scale** | **Baseline** | **Follow-up** | **P value (baseline vs. follow-up)**  (t-tests, unpaired, two-tailed) |
| --- | --- | --- | --- |
| HAMD | 21.8 ± 3.5 | 10 ± 6.3 | <0.001 |
| PANSSpos | 17.6 ± 5.8 | 10.7 ± 5.0 | 0.002 |
| PANSSneg | 20.3 ±5.0 | 8.9 ± 2.5 | <0.001 |
| PANSSgen | 72.7 ± 16.7 | 41.7 ± 10.8 | <0.001 |

Diagnostic groups show a marked decrease in symptom severity from baseline to follow-up.

HAMD = Hamilton Rating Scale for Depression; PANSSpos= positive symptom subscale of the Positive and Negative Symptoms Scale for Schizophrenia; PANSSneg= negative symptom subscale of the PANSS; PANSSgen= general psychopathology subscale of the PANSS
