# Supplementary material for: Slow-wave sleep predicts long-term social functioning in severe mental illness
Source: PLoS One. 2018 Aug 29;13(8):e0202198. doi: 10.1371/journal.pone.0202198 (PMC6114721; doi:10.1371/journal.pone.0202198)
Supplement: S2 Table — A. At baseline. B. At follow-up. Generals psychopathology but neither negative symptoms nor positive symptoms predict social functioning at baseline (A) or follow-up (B). PANSSneg = negative symptom subscale of the PANSS; PANSSpos = positive symptom subscale of the Positive and Negative Symptoms Scale for Schizophrenia; PANSSgen = general psychopathology subscale of the PANSS. Reported are adjusted R2. (DOCX) [file pone.0202198.s003.docx]

| **Model**  Symptom severity  (baseline) | **Variables** | **Standardized beta coefficient** | ***P*-value** |
| --- | --- | --- | --- |
| (R² = 0.325; p < 0.001) | PANSSneg | 0.080 | 0.633 |
|  | PANSSpos | 0.051 | 0.758 |
|  | PANSSgen | -0.589 | **<0.001** |

**S2 Table. Regression models of PANSS-subscores with social functioning as dependent variable.**

**A. At baseline.**

**B. At follow-up.**

| **Model**  Symptom severity (follow-up) | **Variables** | **Standardized beta coefficient** | ***P*-value** |
| --- | --- | --- | --- |
| (R² = 0.290; p = 0.001) | PANSSneg | 0.183 | 0.246 |
|  | PANSSpos | 0.045 | 0.810 |
|  | PANSSgen | -0.560 | **0.001** |

Generals psychopathology but neither negative symptoms nor positive symptoms predict social functioning at baseline (A) or follow-up (B). PANSSneg= negative symptom subscale of the PANSS; PANSSpos= positive symptom subscale of the Positive and Negative Symptoms Scale for Schizophrenia; PANSSgen= general psychopathology subscale of the PANSS. Reported are adjusted R².
