# Supplementary material for: Slow-wave sleep predicts long-term social functioning in severe mental illness
Source: PLoS One. 2018 Aug 29;13(8):e0202198. doi: 10.1371/journal.pone.0202198 (PMC6114721; doi:10.1371/journal.pone.0202198)
Supplement: S3 Table — A. Integrative regression model under exclusion of an outlier in numbers of hospitalizations. Exclusion of the outlier reduces the predictive value of the hospitalizations variable. Slow-wave sleep (SWS) remains a significant predictor. The whole model has a lower R2 than the original integrative regression model (R2 = 0.427 vs. R2 = 0.507). SWS = Slow-wave sleep; VLMT = Rey Auditory-Verbal Learning Test (German version). Reported is the adjusted R2. B. Time to follow-up is added to the integrative regression model as predictor variable. Time to follow-up is not a significant predictor of social functioning. Slow-wave sleep (SWS) remains a significant predictor. The whole model has a lower R2 than the original integrative regression model (R2 = 0.486 vs. R2 = 0.507). SWS = Slow-wave sleep; VLMT = Rey Auditory-Verbal Learning Test (German version). Reported is the adjusted R2. C. Baseline social functioning is added to the integrative regression model as predictor variable. Baseline social functioning is a significant predictor of social functioning. Slow-wave sleep (SWS) remains a significant predictor. The whole model has a higher R2 than the original integrative regression model (R2 = 0.577 vs. R2 = 0.507). SWS = Slow-wave sleep; VLMT = Rey Auditory-Verbal Learning Test (German version). Reported is the adjusted R2. D. Diagnosis is added to the integrative regression model as predictor variable. As in the explorative clincal characteristics model, diagnosis is not a significant predictor of social functioning when added to the integrative regression model. Slow-wave sleep (SWS) remains a significant predictor. The whole model has a lower R2 than the original integrative regression model (R2 = 0.487 vs. R2 = 0.507). SWS = Slow-wave sleep; VLMT = Rey Auditory-Verbal Learning Test (German version). Reported is the adjusted R2. E. Diagnosis is added to the refined regression model including baseline functioning as predictor variable. As in the explorative disease char [file pone.0202198.s004.docx]

**S3 Table. Refining regression models.**

**A. Integrative regression model under exclusion of an outlier in numbers of hospitalizations.**

| **Model** | **Variables** | **Standardized beta coefficient** | ***P*-value** |
| --- | --- | --- | --- |
| Final | SWS | 0.331 | 0.043 |
| (R² = 0.427; p = 0.001) | VLMT | 0.191 | 0.291 |
|  | Hospitalizations | -0.278 | 0.086 |
|  | Symptom severity (baseline) | -0.221 | 0.233 |

Exclusion of the outlier reduces the predictive value of the hospitalizations variable. Slow-wave sleep (SWS) remains a significant predictor. The whole model has a lower R² than the original integrative regression model (R²=0.427 vs. R²=0.507). SWS= Slow-wave sleep; VLMT= Rey Auditory-Verbal Learning Test (German version). Reported is the adjusted R².

**B. Time to follow-up is added to the integrative regression model as predictor variable.**

| **Model** | **Variables** | **Standardized beta coefficient** | ***P*-value** |
| --- | --- | --- | --- |
| Final | SWS | 0.333 | 0.030 |
| (R² = 0.486; p < 0.001) | VLMT | 0.224 | 0.160 |
|  | Hospitalizations | -0.44 | 0.005 |
|  | Symptom severity (baseline) | -0.327 | 0.040 |
|  | Time to follow-up | -0.029 | 0.679 |

Time to follow-up is not a significant predictor of social functioning. Slow-wave sleep (SWS) remains a significant predictor. The whole model has a lower R² than the original integrative regression model (R²=0.486 vs. R²=0.507). SWS= Slow-wave sleep; VLMT= Rey Auditory-Verbal Learning Test (German version). Reported is the adjusted R².

**C. Baseline social functioning is added to the integrative regression model as predictor variable.**

| **Model** | **Variables** | **Standardized beta coefficient** | ***P*-value** |
| --- | --- | --- | --- |
| Final | SWS | 0.280 | 0.046 |
| (R² = 0.577; p < 0.001) | Baseline functioning | 0.378 | 0.033 |
|  | Hospitalizations | -0.482 | 0.005 |
|  | VLMT | 0.091 | 0.561 |
|  | Symptom severity (baseline) | -0.074 | 0.663 |

Baseline social functioning is a significant predictor of social functioning. Slow-wave sleep (SWS) remains a significant predictor. The whole model has a higher R² than the original integrative regression model (R²=0.577 vs. R²=0.507). SWS= Slow-wave sleep; VLMT= Rey Auditory-Verbal Learning Test (German version). Reported is the adjusted R².

**D. Diagnosis is added to the integrative regression model as predictor variable.**

| **Model** | **Variables** | **Standardized beta coefficient** | ***P*-value** |
| --- | --- | --- | --- |
| Final | SWS | 0.315 | 0.044 |
| (R² = 0.487; p = 0.001) | VLMT | 0.224 | 0.169 |
|  | Hospitalizations | -0.481 | 0.004 |
|  | Symptom severity (baseline) | -0.199 | 0.450 |
|  | Diagnosis | 0.012 | 0.961 |

As in the explorative disease characteristics model, diagnosis is not a significant predictor of social functioning when added to the integrative regression model. Slow-wave sleep (SWS) remains a significant predictor. The whole model has a lower R² than the original integrative regression model (R²=0.487 vs. R²=0.507). SWS= Slow-wave sleep; VLMT= Rey Auditory-Verbal Learning Test (German version). Reported is the adjusted R².

**E. Diagnosis is added to the refined regression model including baseline functioning as predictor variable.**

| **Model** | **Variables** | **Standardized beta coefficient** | ***P*-value** |
| --- | --- | --- | --- |
| Final | SWS | 0.288 | 0.048 |
| (R² = 0.561; p < 0.001) | Baseline functioning | 0.387 | 0.034 |
|  | Hospitalizations | -0.431 | 0.005 |
|  | VLMT | 0.088 | 0.583 |
|  | Symptom severity (baseline) | -0.131 | 0.594 |
|  | Diagnosis | -0.078 | 0.743 |

As in the explorative disease characteristics model, diagnosis is not a significant predictor of social functioning when added to the refined integrative regression model also including baseline social functioning. Slow-wave sleep (SWS) remains a significant predictor. The whole model has a lower R² than the refined integrative regression model (R²=0.561 vs. R²=0.577). SWS= Slow-wave sleep; VLMT= Rey Auditory-Verbal Learning Test (German version). Reported is the adjusted R².

**F. Application of the integrative regression model to the depression group only.**

| **Model** | **Variables** | **Standardized beta coefficient** | ***P*-value** |
| --- | --- | --- | --- |
| Final | SWS | 0.400 | 0.253 |
| (R² = -0.182; p = 0.762) | VLMT | -0.032 | 0.930 |
|  | Hospitalizations | -0.279 | 0.423 |
|  | Symptom severity (baseline) | -0.171 | 0.642 |

The integrative regression model is no longer significant, if applied to the depression subgroup alone. SWS= Slow-wave sleep; VLMT= Rey Auditory-Verbal Learning Test (German version). Reported is the adjusted R².

**G. Application of the integrative regression model to the schizophrenia group only.**

| **Model** | **Variables** | **Standardized beta coefficient** | ***P*-value** |
| --- | --- | --- | --- |
| Final | SWS | 0.460 | 0.048 |
| (R² = 0.514; p = 0.021) | VLMT | 0.482 | 0.057 |
|  | Hospitalizations | -0.790 | 0.004 |
|  | Symptom severity (baseline) | 0.044 | 0.845 |

Application of the integrative regression model to the schizophrenia subgroup results in a significant model with SWS and no. of hospitalizations as significant predictors. Verbal memory tends towards significance. SWS= Slow-wave sleep; VLMT= Rey Auditory-Verbal Learning Test (German version). Reported is the adjusted R².
